# Validity of cingulate–precuneus–temporo-parietal hypometabolism for single-subject diagnosis of biomarker-proven atypical variants of Alzheimer’s Disease

**DOI:** 10.1007/s00415-022-11086-y

**Published:** 2022-03-26

**Authors:** Valeria Isella, Cinzia Crivellaro, Anna Formenti, Monica Musarra, Sara Pacella, Sabrina Morzenti, Francesca Ferri, Cristina Mapelli, Francesca Gallivanone, Luca Guerra, Ildebrando Appollonio, Carlo Ferrarese

**Affiliations:** 1 Neurology Department, San Gerardo Hospital, University of Milano – Bicocca, Monza, Italy; 2 Nuclear Medicine Unit, San Gerardo Hospital, University of Milano - Bicocca, Monza, Italy; 3 Medical Physics, San Gerardo Hospital, Monza, Italy; 4 Institute of Molecular Bioimaging and Physiology, National Research Council (IBFM-CNR), Segrate, Milan, Italy; 5 Neurology Department, School of Medicine and Surgery, University of Milano - Bicocca, Via Cadore 48, 20900 Monza, MB Italy

**Keywords:** FDG-PET, Alzheimer’s Disease, Brain metabolism, Biomarkers, Dementia

## Abstract

The aim of our study was to establish empirically to what extent reduced glucose uptake in the precuneus, posterior cingulate and/or temporo-parietal cortex (PCTP), which is thought to indicate brain amyloidosis in patients with dementia or MCI due to Alzheimer’s Disease (AD), permits to distinguish amyloid-positive from amyloid-negative patients with non-classical AD phenotypes at the single-case level. We enrolled 127 neurodegenerative patients with cognitive impairment and a positive (n. 63) or negative (n. 64) amyloid marker (cerebrospinal fluid or amy-PET). Three rating methods of FDG-PET scan were applied: purely qualitative visual interpretation of uptake images (VIUI), and visual reading assisted by a semi-automated and semi-quantitative tool: INLAB, provided by the Italian National Research Council, or Cortex ID Suite, marketed by GE Healthcare. Fourteen scans (11.0%) patients remained unclassified by VIUI or INLAB procedures, therefore, validity values were computed on the remaining 113 cases. The three rating approaches showed good total accuracy (77–78%), good to optimal sensitivity (81–93%), but poorer specificity (62–75%). VIUI showed the highest sensitivity and the lowest specificity, and also the highest proportion of unclassified cases. Cases with asymmetric temporo-parietal hypometabolism and a progressive aphasia or corticobasal clinical profile, in particular, tended to be rated as AD-like, even if biomarkers indicated non-amyloid pathology. Our findings provide formal support to the value of PCTP hypometabolism for single-level diagnosis of amyloid pathophysiology in atypical AD, but also highlight the risk of qualitative assessment to misclassify patients with non-AD PPA or CBS underpinned by asymmetric temporo-parietal hypometabolism.

**Supplementary Information:**

The online version contains supplementary material available at 10.1007/s00415-022-11086-y.

## Introduction

Research criteria for the diagnosis of Alzheimer’s Disease (AD), in the Mild Cognitive Impairment (MCI) or dementia stages [1–3], incorporate biomarkers in the diagnostic process, converging on the recommendations about the use of the so-called *pathophysiological* biomarkers, namely Positron Emission Tomography with ligands for cerebral amyloid deposits (amy-PET) and Aβ, Tau and phospho-Tau in cerebrospinal fluid (CSF), but diverging on the role of brain 18-fluorodeoxy-glucose PET (FDG-PET). According to the National Institute of Neurological and Communicative Disorders and Stroke and Alzheimer’s Disease and Related Disorders Association (NINCDS-ADRDA) [2], in the absence of markers of Aβ deposits, a specific FDG-PET pattern, i.e. hypometabolism in the Precuneus, posterior Cingulate and posterior Temporo-Parietal (PCTP) regions, may be used as a marker of an underlying AD pathophysiology. On the other hand, the International Working Group (IWG) [3] classifies PCTP hypometabolism as a *topographical* marker, which maps spreading of neurodegeneration but lacks sufficient pathophysiological specificity.

The link between AD and PCTP hypometabolism has received robust support in the literature, crucially also from case reports and group studies including AD patients with post-mortem or in vivo positive markers of amyloidopathy [4–16]. These regions are highly interconnected and hyperactive hubs of the default mode network, and seem to be particularly vulnerable to Aβ aggregation due to their high metabolic demands [17]. Remarkably, PCTP hypometabolism has been demonstrated not only in the classical, amnestic, type of AD, but also in its atypical presentations, i.e. the frontal [13, 14], linguistic [13–16] and visuospatial [7, 13–15] variants. However, only a limited number of studies have so far assessed the validity of PCTP hypometabolism as an index of amyloidopathy at the single patient-level in samples with a neuropathological or biomarker-based diagnosis of AD [18–23]; moreover, only one study [24] considered a mixed, amnestic and atypical, pool of amy-positive (A+) and amy-negative (A − ) patients.

The current study was designed to fill this literature gap and test the validity of PCTP hypometabolism for single-subject diagnosis of Aβ-related cognitive deficits in a specific clinical scenario: diagnostic work up of referrals to a tertiary memory clinic, with a focus on atypical presentations of AD, in various disease stages. All patients had undergone amy-PET or CSF as pathophysiological biomarkers, which were used as gold standard for a clinico-biological diagnosis of AD.

Recent consensus recommendations from the European Association of Nuclear Medicine and European Academy of Neurology (EANM-EAN) [25], emphasised the usefulness of semi-automated processing to assist visual reading, thus we also compared the validity of PCTP assessed with a purely qualitative approach, consisting in the visual interpretation of uptake images (VIUI), with two automated tools that allow semi-quantification of FDG-uptake and comparison with an age-matched database of healthy controls.

## Materials and methods

### Participants

We reviewed medical records (incorporating neurological examination, MiniMental State Examination—MMSE—score and neuroimaging reports) of subjects referred for cognitive disturbances to the memory clinic of San Gerardo Hospital, Monza, between January 2015 and January 2020. Criteria for eligibility were the following: (1) diagnosis of dementia or MCI according to NINCDS-ADRDA criteria, or of preclinical AD defined as an asymptomatic condition, at the time of FDG-PET, with biomarker evidence of AD pathology, or converted to MCI/dementia at follow-up [1–3]; (2) availability of brain FDG-PET scan performed as part of the routine diagnostic work up within six months from neurological assessment; (3) availability of a physiopathological biomarker for amyloid status (amyloid-PET or Tau/Aβ ratio in CSF) performed for research purposes, or presence of a genetic mutation for a neurodegenerative disorder. Individual, syndrome-level diagnoses did not take into account the results of brain FDG-PET scan and were based on standardised criteria for amnestic AD [2], Posterior Cortical Atrophy (PCA) [26], behavioural variant Frontotemporal Dementia (bFTD) [27], Dementia with Lewy Bodies (DLB) [28], Corticobasal Syndrome (CBS) [29], Primary Progressive Aphasia (PPA) [30], or Progressive Supranuclear Palsy (PSP) [31].

Exclusion criteria were evidence of moderate-to-severe vascular burden on structural neuroimaging or history of other neurological disorders, major psychiatric diseases, brain injury, mental insufficiency, substance abuse, severe medical conditions.

All participants were unpaid volunteers and gave written informed consent for participation. The study was approved by our institution's ethics committee, Comitato Etico Brianza, and was carried out in accordance with the ethical standards of 1964 Declaration of Helsinki and its later amendments.

### Acquisition and processing of FDG-PET scans

All scans were acquired with the same General Electric Discovery LS PET/CT scanner in our Nuclear Medicine Unit, within 6 months from the neurological assessment.

Patients were instructed to fast for at least 6 hours. Before the exam, they were measured blood glucose levels (whole sample’s mean levels: 105.0 ± 19.6 mg/dL), and then received an intravenous bolus of approximately 200 MBq of 18F-FDG. After lying supine in a quiet, dimly lit room for approximately 45 min, they were transferred to the scanner. First, a CT scan was performed for attenuation correction, then PET images were acquired for 15 min, with a thickness of 3.27 mm and a matrix of 128 × 128 pixels. Subsequent image reconstruction followed an ordered subset expectation maximisation (OSEM) algorithm.

### Processing of PET images with INLAB

**Fig. 1:**
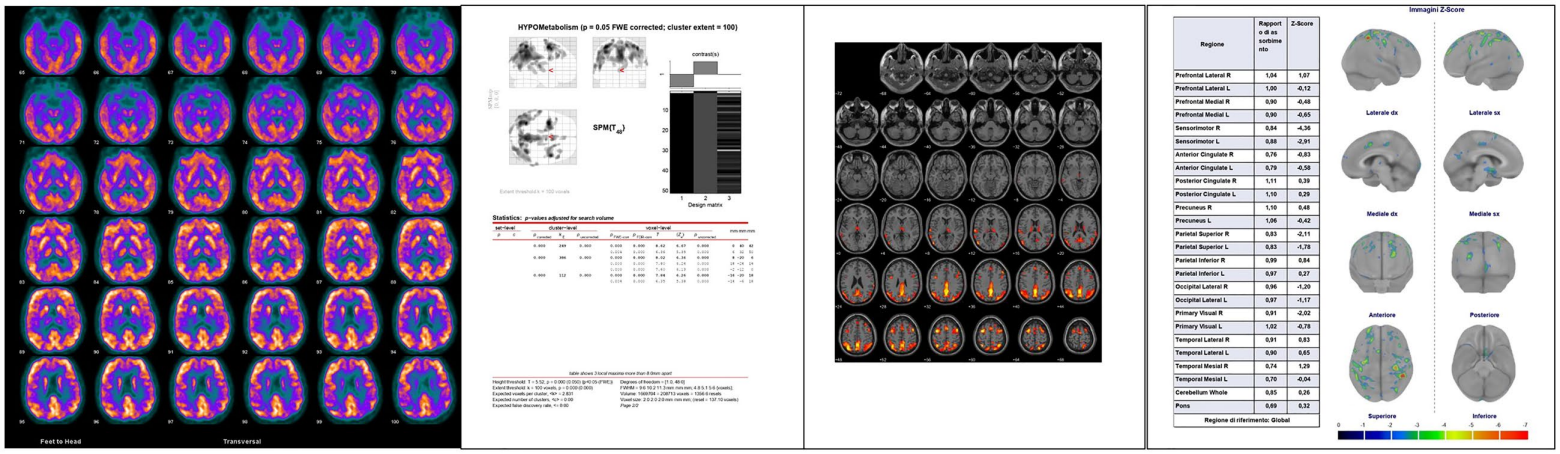
Examples of output from the three FDG-PET scan rating methods for an amyloid-positive patient with an amnestic presentation (from left to right): display for visual interpretation of uptake images; INLAB glass brain and statistical map; output of Cortex ID Suite

INLAB is a validated automated service for analysis of PET images [32] based on Statistical Parametric Mapping (SPM; http://www.fil.ion.ucl.ac.uk/spm) developed by the Bioimaging Lab of the Italian National Research Council (https://www.ibfm.cnr.it/), and freely available online. Individual patients’ PET scans are uploaded as DICOM files archived together in.zip format. They are reoriented along the anterior–posterior commissure, spatially normalised to an FDG-PET dementia-specific template [33], smoothed with an isotropic 3D Gaussian kernel of 8 mm FWHM, and proportionally scaled to a whole-brain mean intensity value. Pre-processed images are then tested for relative hypometabolism by comparison with a reference group of 112 healthy controls [34], using the two sample t-test design of SPM5, including age as covariate. Cluster-level significance threshold is set at *p* < 0.05 FWE-corrected, and only clusters with a minimum size of 100 voxels are retained. Areas of significant hypometabolism are finally displayed as areas of black and white shading on a three-orientation ‘glass brain’, as well as in a colour statistical parametric map. A table reporting set-, cluster- and voxel-level statistics and anatomical coordinates of significant clusters is also produced. All outputs can be downloaded as .pdf documents (Fig. 1).

### Processing of PET images with Cortex ID Suite

Cortex ID Suite is a software package developed and marketed by GE Healthcare (Waukesha, WI, USA) that computes individual patients’ mean FDG-uptake for cerebellum and pons, also used as reference regions for scaling, and 12 supratentorial, bilateral regions of interest (ROI): lateral and mesial prefrontal cortex, anterior and posterior cingulate, sensorimotor cortex, precuneus, superior and inferior parietal lobe, lateral and mesial temporal cortex, lateral occipital cortex, and primary visual cortex. Patients’ mean values are compared with those of an inbuilt dataset of scans from 294 healthy controls. Results of this comparison are shown in a colour map projected on a 3D stereotactic surface, with manipulable colour scale, and also expressed as Z-scores displayed in a table, with a Z-score ≤  − 2.0 as cutoff for significant hypometabolism. All outputs can be downloaded as .pdf documents (Fig. 1).

### Rating of PET images

PET images were all assessed by one rater, M.M., a Nuclear Medicine specialist with over 20 years of experience reading PET scans, who was blinded to clinical diagnosis and biomarker status. For the VIUI rating procedure, a second, independent, rater, S.P., a last-year resident in Nuclear Medicine, was involved, with the aim to evaluate inter-rater reliability between two assessors with different levels of expertise.

VIUI was based solely on visual inspection of qualitative images, which were displayed on a terminal on which orientation (axial, coronal, and sagittal) and colour scale could be manipulated (Fig. 1). INLAB rating was based on visual inspection of colour statistical parametric maps and glass brain images, and on semi-quantitative indices provided by SPM. Cortex ID Suite rating was based on visual inspection of 3D colour maps and on the Z-scores computed by the software.

The rater was asked to focus on PCTP regions, rate them as normal/hypometabolic/unclassifiable, and made a judgement about whether or not the observed pattern reflected a diagnosis of AD. Specifically, following criteria similar to those applied in previous studies [21, 22, 35], images consistent with AD were agreed upon a priori to show hypometabolism in the precuneus/posterior cingulate *and/or* temporo-parietal regions, either restricted to these areas, or clearly predominant in these areas than in the frontal, fronto-parietal or occipital regions.

The three ratings (VIUI and evaluation of INLAB and Cortex ID Suite outputs) were performed in three distinct sessions, blinded to the results of prior ratings.

### Statistical analysis

Statistical analysis was performed with SPSS version 27.0 (IBM Corp., Armonk, NY, USA) or MedCalc Statistical Software version 20.027 (MedCalc Software bv, Ostend, Belgium; https://www.medcalc.org; 2020).

Comparisons between A+ and A − cases were carried out with Student’s *t*-test for continuous variables (age, disease duration, MMSE score) and Chi-square test for categorical variables (sex), setting threshold for significance at *p* < 0.05.

Total accuracy, sensitivity, specificity and positive and negative predictive values (PPV, NPV) were calculated as measures of the validity of PCTP hypometabolism to classify correctly A+ and A − cases. They were computed for each of the three PET scans rating methods. Positive and negative Likelihood Ratios (+ LR, − LR) were also calculated, with the formulas: sensitivity/(100—specificity), (100—sensitivity)/specificity. A +LR value > 1 means that a positive test is more likely to occur in patients with the condition (i.e. Aβ pathology) than in those without the condition, while a − LR value < 1 means that a negative test is more likely to occur in patients without the condition than in those with the condition. Differences in accuracy between the three rating methods were tested for statistical significance by computing receiver-operating characteristic (ROC) curves and comparing the areas under the curve (AUC) using DeLong et al. methodology [36], in MedCalc.

Fleiss kappa was run to determine the degree of concordance between the three rating methods, and Cohen’s kappa was run to measure inter-rater reliability for VIUI.

## Results

### Characteristics of the study sample

**Fig. 2:**
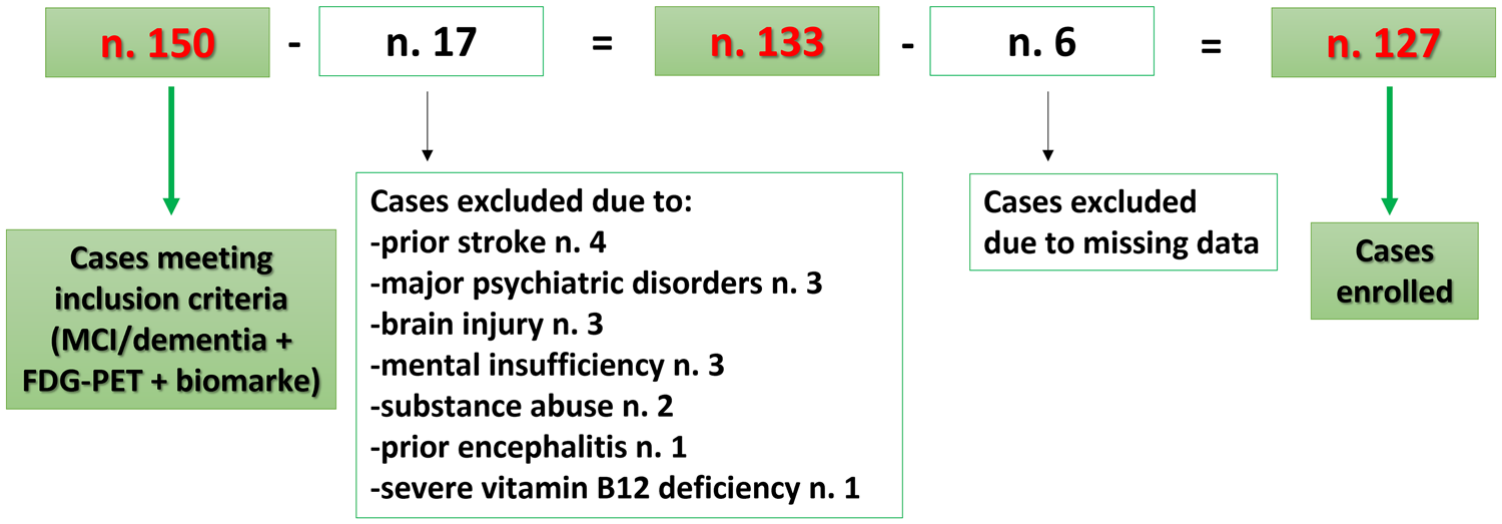
Flowchart of patients enrolment in the study

From an initial pool of 150 patients meeting inclusion criteria, we excluded 23 cases due to the reasons detailed in Fig. 2.

The final study sample was, therefore, composed by 127 patients, whose general features are shown in Table 1. They all met criteria for MCI or dementia, except three, who were in a preclinical disease stage: all three complained of word finding difficulties at the time of FDG-PET, and later converted to PPA (n. 2) or (CBS) (n. 1). Sixty-three out of 127 (49.6%) were A+ and 64 A − (Table 1). The two groups showed overlapping age (*t* = 0.014, *p* = 0.989) and sex distribution (*x*_2_ = 0.378, *p* = 0.593); mean MMSE score (*t* = − 1.916, *p* = 0.058) and disease duration (*t* = 1.859, *p* = 0.066) were marginally not significant. CSF (with Tau/Aβ ratio ≥ 1.0 as cutoff for amyloid positivity [37]) was the most frequent biomarker, being available in 94/127 (74.0%) cases (40 A+ and in 54 A − ). There were only three cases of genetic mutations: one Presenilin 1 and one Presenilin 2 presenting as PCA, and one Progranulin presenting with predominant language deficits. The remaining 30 patients (19 A+ and 11 A − ) had PET with amyloid tracer as biomarker ([^18^F]Flutemetamol in 13 cases, [^18^F]Florbetapir in 12, and [^18^F]Florbetaben in 10, analysed qualitatively in most cases). Within the A+ group, the amnestic and PCA presentations accounted for more than half of all cases, while the most prevalent phenotypes within the A − group were CBS and PPA, followed by bFTD (overall significance for comparison of proportion of the different syndromes between the two groups: *x*_2_ = 42.372, *p* = 0.0001). Within each clinical syndrome, the proportion of A+ patients was as follows: 9/26 PPA (34.6%), 9/25 CBS (36.0%), 20/24 amnestic AD (83.3%), 17/18 PCA (94.4%), 4/15 bFTD (26.7%), 1/6 DLB (16.7%), 1/5 not otherwise specified syndrome (20.0%), 0/5 PSP, and 2/3 subjective complaints (66.7%).

**Table 1:** Demographic and clinical characteristics of the study cohort

	Total sample	Amyloid-positive	Amyloid-negative
	n. 127	n. 63	n. 64
Age	69.3 ± 8.3	69.3 ± 8.9	69.3 ± 7.8
Sex—n. men (%)	72 (56.7%)	34 (54.0%)	38 (59.4%)
MiniMental State Examination	22.9 ± 5.2	22.0 ± 5.4	23.8 ± 4.9
Years from onset to FDG-PET	2.5 ± 1.6	2.8 ± 1.7	2.3 ± 1.5
Clinical syndrome—n. (%):			
Primary progressive aphasia	26 (20.5%)	9 (14.3%)	17 (26.6%)
Corticobasal syndrome	25 (19.7%)	9 (14.3%)	16 (25.0%)
Amnestic presentation	24 (18.9%)	20 (31.7%)	4 (6.2%)
Posterior cortical atrophy	18 (14.2%)	17 (27.0%)	1 (1.6%)
Behavioural fronto-temporal dementia	15 (11.8%)	4 (6.3%)	11 (17.2%)
Lewy body disease	6 (4.7%)	1 (1.6%)	5 (7.8%)
Not otherwise specified	5 (3.9%)	1 (1.6%)	4 (6.2%)
Progressive supranuclear palsy	5 (3.9%)	0	5 (7.8%)
Subjective cognitive complaints	3 (2.4%)	2 (3.2%)^a^	1 (1.6%)^b^

All values are means ± standard deviation, unless otherwise stated

^a^Later diagnosed one with corticobasal syndrome and the other with primary progressive aphasia

^b^ Later diagnosed with primary progressive aphasia

### Results of analyses on FDG-PET ratings

In 14 out of 127 patients (11.0%), PET scans could not be rated as positive or negative by VIUI (n. 9), on INLAB images (n. 1), or by both VIUI and INLAB procedures (n. 4), while Cortex ID Suite allowed to reach a clear-cut conclusion in all cases. Their main demographic, clinical and PET imaging features are reported in the Supplementary material. Most (11/14, 78.6%) were A − patients for which the two raters could not rule out significant PCTP hypometabolism, and approximately half of them showed impairment of language.

Inter-rater reliability for VIUI was calculated on all 127 cases, while validity values were calculated on the 113 patients whose scans could be rated as either normal or hypometabolic.

### Inter-rater reliability for VIUI

Cohen’s k indicated high agreement between the two Nuclear Medicine physicians in defining presence/absence of PCTP hypometabolism (0.83 [95% CI 0.70 to 0.97], *p* = 0.000).

There were 11 discordant ratings: four scans were rated as AD-like by M.M. and as negative by S.P., three were rated as AD-like by S.P. and as negative by M.M., and four were rated as AD-like (two) or negative (two) by M.M. and as unclassifiable by S.P.

### Agreement between the three rating methods

VIUI showed moderate agreement with both rating of INLAB maps and rating of Cortex ID Suite maps (Table 2). In most of the 21 discrepant cases, PCTP regions were rated as hypometabolic on VIUI and as normal in INLAB images (n. 16, 88.9%) and Cortex ID Suite images (n. 14, 66.7%).

**Table 2:** Results of Fleiss kappa analysis for the three FDG-PET rating procedures

INLAB	VIUI	
	Positive	Negative
Positive	59	2
Negative	16	36
Agreement: 84.1%		
κ = .67 [95% CI, .60 to .74] (p = .000)		

Cortex ID Suite	VIUI	
	Positive	Negative
Positive	61	7
Negative	14	31
Agreement: 81.4%		
κ = .60 [95% CI, .52 to .68] (p = .000)		

Cortex ID Suite	INLAB	
	Positive	Negative
Positive	59	9
Negative	2	43
Agreement: 90.3%		
κ = .80 [95% CI, .74 to .86] (p = .000)		

*VIUI*  visual interpretation of uptake images

Between the two automated methods, agreement was strong (Table 5), and the majority of the 11 discrepancies (n. 9, 81.8%) were scans rated as AD-like on Cortex ID Suite output and as normal on INLAB output.

### Results of validity analysis

Total accuracy of PCTP hypometabolism in classifying A+ and A − cases was around 77% for all three rating procedures (Table 3). Sensitivity and NPV were generally high (ranging from 81 to 93% and from 79 to 89%, respectively), while specificity and PPV were generally lower (ranging from 62 to 75% and from 72 to 77%, respectively). +LR ranged from 2.4 to 3.2, and − LR from 0.25 to 0.11. VIUI showed the highest sensitivity, NPV and − LR, while rating of INLAB images showed the highest specificity, PPV and +LR.

**Table 3:** Validity in identifying amyloid-positive and amyloid-negative patients of hypometabolism at the level of precuneus, posterior cingulate and temporo-parietal regions

	VIUI	INLAB	Cortex ID Suite
n. True positive	54	47	50
n. True negative	34	41	37
n. False positive	21	14	18
n. False negative	4	11	8
Sensitivity	93.1%	81.0%	86.2%
Specificity	61.8%	74.6%	67.3%
Positive predictive value	72.0%	77.1%	73.5%
Negative predictive value	89.5%	78.9%	82.2%
Total accuracy	77.9%	77.9%	77.0%
Positive likelihood ratio	2.44	3.18	2.63
Negative likelihood ratio	0.11	0.25	0.21

Values are displayed for all three rating methods

*VIUI* visual interpretation of uptake images

**Fig. 3:**
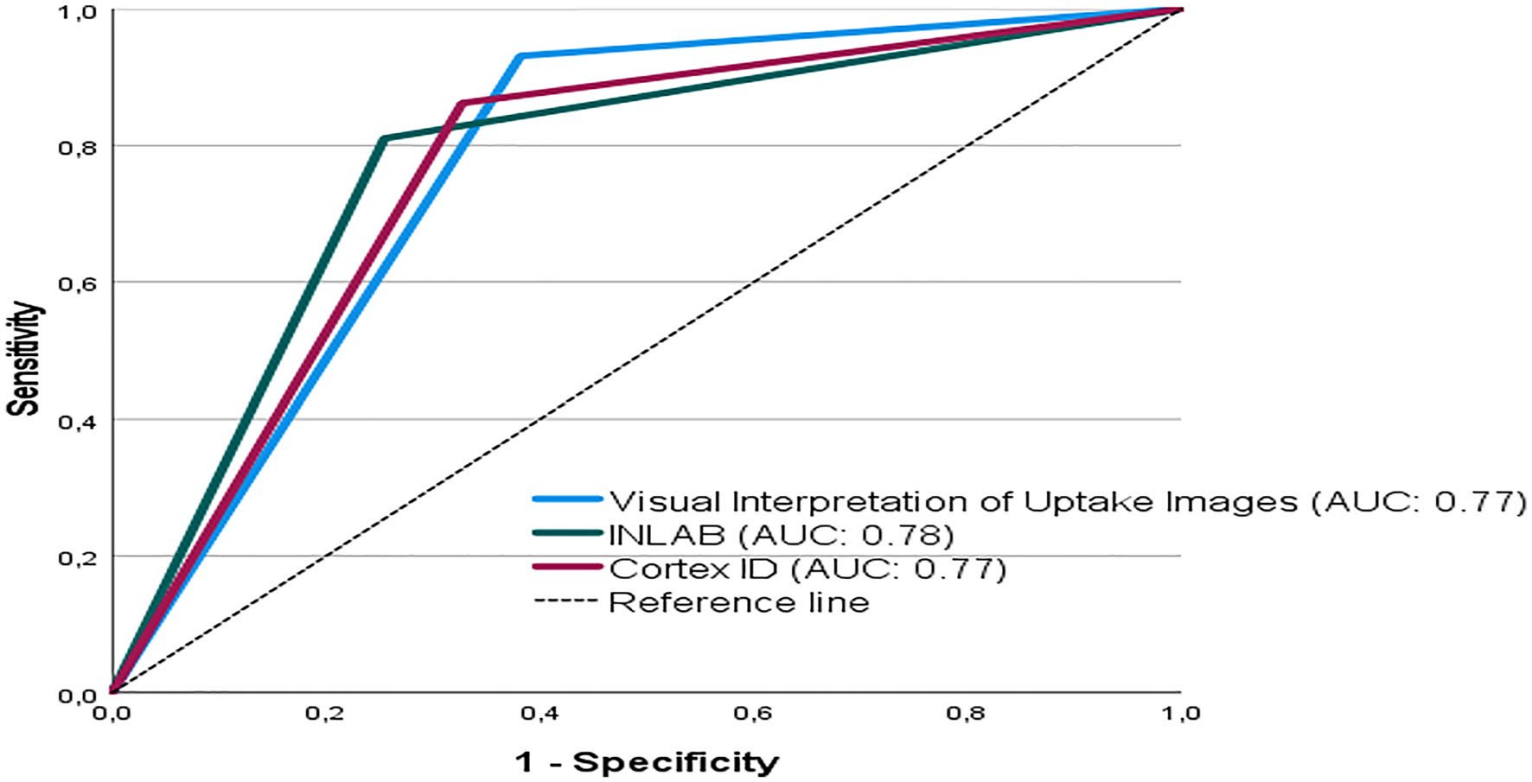
Receiver-operating characteristic curves and areas under the curve (AUC) for the three rating methods

Comparison of AUCs between the three rating methods (Fig. 3) did not show significant differences (*p* = 0.9277 between VIUI and INLAB, *p* = 0.8611 between VIUI and Cortex ID, *p* = 0.7182 between INLAB and Cortex ID).

### Characteristics of misclassified cases

A total of 40 patients were misclassified by one or more of the three rating methods: 28 were false positive, i.e. A − cases whose PCTP regions were rated as hypometabolic, and 12 were false negative, i.e. A+ cases without evidence of significant PCTP hypometabolism.

Table 4 shows the main characteristics of the 12 false-negative cases, seven of which were false negative for two or more rating methods. These patients were overall relatively old: 11/12 (91.7%), were above 70 years of age and their mean age (74.5 years ± 3.1) was 5 years older than the entire study sample’s. They also were in an initial disease stage: eight (66.7%) showed a maximum symptoms duration of 2 years, and seven (58.3%) had an MMSE score ≥ 26/30. Finally, they showed diverse clinical presentations, but the amnestic phenotype was slightly prevalent (n. 5, 41.7%).

**Table 4:** Individual demographic and clinical characteristics of amyloid-positive cases without significant precuneus, posterior cingulate and temporo-parietal (PCTP) hypometabolism (false negative, FN)

	Age	Syndrome	Months onset-PET	MMSE	Biomarker	Tau/Aβ ratio	Summary of hypometabolic regions
FN1^abc^	73	SD	12	28	PET	–	L > R Basal temporal cortex
FN2^abc^	74	DLB	30	23	PET	–	BIL insular and occipito-parietal cortex
FN3^abc^	76	AMN	24	26	CSF	3.08	R > L fronto-temporal cortex
FN4^abc^	76	bFTD	24	16	CSF	1.75	R fronto-temporal cortex
FN5^bc^	73	AMN	12	26	CSF	2.32	BIL frontal cortex
FN6^bc^	77	PCA	12	26	PET	–	BIL fronto-parietal cortex
FN7^bc^	73	PCA	48	27	CSF	2.75	BIL fronto-parietal, occipital and mesial temporal cortex
FN8^b^	76	CBS	16	28	CSF	1.62	L > R fronto-parietal cortex
FN9^b^	73	AMN	48	22	PET	–	L fronto-temporal cortex
FN10^b^	78	AMN	36	27	CSF	1.41	R mesial temporal cortex
FN11^b^	78	NOS PPA	24	18	CSF	1.65	L > R Ant cing, left par, mes occipital
FN12^c^	67	AMN	12	24	CSF	1.42	L fronto-parietal and occipital cortex

*AMN* amnestic, *bFTD* behavioural fronto-temporal dementia, *BIL* bilateral, *CSF* cerebrospinal fluid, *CBS* corticobasal syndrome, *L* left, *DLB* dementia with Lewy bodies, *MMSE* MiniMental State Examination, *NOS* not otherwise specified, *PCA* posterior cortical atrophy, *PPA* primary progressive aphasia, *R* right, *SD* semantic dementia

Misclassified by ^a^visual interpretation of uptake images

^b^INLAB

^c^Cortex ID Suite

In all eight misclassified cases with CSF as biomarker, the Tau/Aβ ratio was well above cutoff for amyloid positivity. As to FDG-PET scans, most patients (n. 8, 66.7%) showed hypometabolism in the fronto-temporal or fronto-parietal regions.

Table 5 displays the main characteristics of the 28 cases misclassified as false positive, half of which were false positive for two or more rating methods. Fifteen (53.4%) were below 70 years of age, and 11 (39.3%) had a pre-senile onset. The great majority were in an early disease stage or were only mildly impaired (n. 20, 71.4%, had an MMSE score ≥ 24, and n. 24, 85.7%, a score ≥ 20). Two phenotypes were prevalent: 11 patients (39.3%) had CBS and 8 (28.6%) PPA; the next more frequent diagnosis was DLB (n. 4, 14.3%). Notably, in addition to the cases of progressive aphasia, six more patients, five with CBS and the patient with the Progranulin mutation, also showed remarkable language deficits. The great majority of these patients (n. 21, 75.0%) had CSF as biomarker. Only in one case (FP22) was the Tau/Aβ ratio close to the cutoff for amyloid positivity.

**Table 5:** Individual demographic and clinical characteristics of amyloid-negative cases with significant precuneus, posterior cingulate and temporo-parietal (PCTP) hypometabolism (false positive, FP)

	Age	Syndrome	Months onset-PET	MMSE	Biomarker	Tau/Aβ ratio	Summary of hypometabolic regions	
							Outside PCTP	Within PCTP
FP1^abc^	73	CBS^d^	48	26	PET	–	L > R fronto-temporal cortex	L > R all areas
FP2^abc^	76	CBS	12	26	CSF	0.78	L > R fronto-temporal cortex	L precuneus, L > R Temporo-parietal cortex
FP3^abc^	64	CBS	48	25	CSF	n.a	–	Precuneus/cingulate, R > L parietal cortex
FP4^abc^	55	CBS^d^	18	23	CSF	0.14	L > R frontal cortex	L > R precuneus and temporo-parietal cortex
FP5^abc^	79	CBS	12	20	CSF	0.51	BIL frontal cortex	Precuneus/cingulate, BIL parietal cortex
FP6^abc^	74	DLB	42	15	CSF	0.45	–	BIL all areas
FP7^abc^	67	AMN	18	21	CSF	0.38	BIL frontal cortex	BIL all areas
FP8^abc^	73	AMN	72	26	CSF	0.79	–	Precuneus/cingulate, L > R parietal cortex
FP9^abc^	70	Logopenic PPA	48	25	CSF	0.85	–	L > R all areas
FP10^abc^	68	NOS PPA	24	30	CSF	0.11	L inferior temporal cortex	L temporo-parietal cortex
FP11^abc^	68	Progranulin^d^	48	15	Genetics	–	L fronto-temporal cortex	L temporo-parietal cortex
FP12^ab^	58	CBS^d^	12	27	CSF	0.26	BIL frontal cortex	Precuneus/cingulate, R > L parietal cortex
FP13^bc^	77	SD	24	25	PET	–	L > R fronto-temporal cortex	Precuneus/cingulate, L > R parietal cortex
FP14^bc^	61	CBS	12	25	CSF	0.17	L frontal cortex	Precuneus/cingulate, L parietal cortex
FP15^a^	69	CBS	48	26	PET	–	–	BIL temporo-parietal cortex
FP16^a^	66	CBS^d^	18	25	CSF	0.52	–	L > R temporo-parietal cortex
FP17^a^	66	CBS^d^	36	8	CSF	0.32	–	L > R temporo-parietal cortex
FP18^a^	66	SD	36	29	PET	–	L > R frontal cortex	L > R temporo-parietal cortex
FP19^a^	71	SD	24	24	PET	–	L > R fronto-temporal cortex	L > R temporo-parietal cortex
FP20^a^	66	LBD	36	11	CSF	0.49	BIL frontal cortex	BIL temporo-parietal cortex
FP21^a^	72	bFTD	12	26	CSF	0.27	BIL frontal cortex	Precuneus, BIL temporo-parietal cortex
FP22^a^	63	SD	24	28	CSF	0.96	BIL fronto-temporal cortex	BIL temporo-parietal cortex
FP23^a^	68	SD	12	30	CSF	0.24	BIL frontal cortex	L temporo-parietal cortex
FP24^c^	56	NOS PPA	12	25	CSF	0.57	L > R frontal cortex	Precuneus/cingulate, L > R parietal cortex
FP25^c^	79	bFTD	48	27	PET	–	L fronto-temporal cortex	Precuneus/cingulate, L parietal cortex
FP26^c^	82	LBD	24	27	CSF	0.13	R > L fronto-temporal and occipital cortex	Precuneus/cingulate, R > L parietal cortex
FP27^c^	77	LBD	12	25	CSF	0.37	BIL occipito-temporal cortex	Precuneus/cingulate, BIL parietal cortex
FP28^c^	73	CBS	12	22	CSF	0.21	L > R frontal cortex	L > R all areas

n.a. not available (results of CSF analysis were reported as ‘indicative of Alzheimer’s Disease’ but no numerical value was provided)

*AMN *Amnestic, *bFTD* behavioural fronto-temporal dementia, *BIL *bilateral, *CSF* cerebrospinal fluid, *CBS* corticobasal syndrome, *L* left, *LBD* Lewy body disease, *MMSE* MiniMental State Examination, *NOS* not otherwise specified, *PCA* posterior cortical atrophy, *PPA* primary progressive aphasia, *R* right, *SD* semantic dementia

Misclassified by: ^a^visual interpretation of uptake images

^b^INLAB

^c^Cortex ID Suite

^d^Predominant impairment of speech

With regard to FDG scans, in most of these patients, hypometabolism within the PCTP system encompassed the temporo-parietal carrefour (n. 13, 46.4%) or the precuneus/posterior cingulate and the parietal cortex (n. 10, 35.7%), and was asymmetric (n. 20, 71.4%), with a left-hemisphere predominance (n. 17, 60.7%). Additional clusters of reduced FDG-uptake were reported in the frontal or fronto-temporal regions in the majority of patients (n. 19, 67.9%); only in seven cases (25.0%), no other area of hypometabolism was evident outside PCTP.

## Discussion

FDG-PET neuroimaging is considered an essential part of the diagnostic algorithm for dementia [25, 38, 39]. In this study, we investigated the role of hypometabolism in PCTP areas in predicting the presence of Aβ pathology at the individual level, in patients with atypical MCI or dementia whose amyloid status was established by CSF, amy-PET, or presence of a genetic mutation. Hypometabolism was assessed through three different approaches: purely qualitative VIUI, and visual + semi-quantitative assessment based on either INLAB or Cortex ID Suite softwares. The three procedures all showed good (77–78%) total accuracy, good to optimal sensitivity (81 to 93%), but poorer specificity (62 to 75%), in agreement with the results from prior studies that converged in showing high sensitivity (85–94%) but more diverse specificity (50–83%) [18–20, 23, 24]. VIUI, which is clinically the most used approach, also showed a good agreement between the expert rater and the Nuclear Medicine resident, indicating that the procedure does not require extremely advanced skills.

The generally high sensitivity of PCTP hypometabolism for amyloid positivity in classical as well as atypical presentations of AD (more than 68% of A+ cases had a non-amnestic syndrome) is in agreement with prior structural and functional neuroimaging studies showing that the involvement of these regions is a common feature of AD, irrespective of the clinical phenotype [6, 7, 13–16, 40, 41], and further supports the idea that this metabolic signature of AD mirrors disease pathophysiology over and above symptoms profile. VIUI, in particular, showed higher sensitivity than INLAB and Cortex ID Suite maps and computations. This finding comes as a confirmation of past evidence suggesting that the overall superiority of semi-quantitative over qualitative reading does not relate to sensitivity, rather to specificity, and varies greatly across tools [8, 25, 42, 43]. Morbelli et al. [43] posited that trained readers might be able to detect minor but meaningful abnormalities that do not reach the threshold for significance on statistical maps. As an example, readers have the possibility to base their evaluation on inter-hemispheric asymmetries, which only few automated tools allow to compute [43].

Scrutiny of socio-demographic and clinical features of our 12 false-negative cases highlighted some relatively consistent features (e.g. they were generally old and in an early disease stage), but was not helpful in identifying clear-cut reasons for their misclassification. Certain characteristics of our 28 false-positive cases, on the other hand, provide an at least partial account for the lower specificity found for PCTP hypometabolism. The majority of these patients, whose biomarkers suggested non-amyloid pathophysiology but who were regarded as having an AD-like FDG pattern, had a diagnosis of CBS (some with predominant language disturbances) or PPA, and showed hypometabolism involving the parietal, temporo-parietal and/or temporal areas, quite often with an asymmetric distribution. In PPA, the poor specificity of quantitative and qualitative FDG-PET in predicting underlying pathology, especially in cases with asymmetric PCTP hypometabolism, had already been pointed out [6, 44, 45]. Altogether, these features suggest that the rater did not fail in judging presence or absence of significant PCTP hypometabolism, rather misinterpreted true PCTP hypometabolism as an index of amyloid pathology, while it was a correlate of the clinical syndrome. In fact, some of the symptoms typical of CBS or of (at least some subtype of) PPA, such as limb apraxia, spatial deficits, or impairment of speech, show degeneration of the parietal and temporal cortex [46–52].

An additional characteristic common to several false-positive cases was the presence of quite diffuse FDG abnormalities, both within and outside the PCTP system (in particular at the level of the frontal lobes). This suggests that in these cases, the areas of hypometabolism typical for AD might be involved secondarily to the spreading of neurodegeneration from the networks initially targeted by non-AD pathology. For instance, six false-positive PPAs were patients with semantic dementia in whom the typical basal temporal abnormalities appeared to extend to the posterior temporal and inferior parietal cortex.

Specificity was particularly poor for purely qualitative VIUI (the major number of—false–—positives was in fact the main reason for the relatively poor agreement between this procedure and the two semi-quantitative ratings). Interestingly, VIUI also showed the highest proportion of unclassified cases, who were for the most part A − patients in whom raters could not disambiguate doubtful PCTP abnormalities. As established by EANM-EAN Delphi round [25], in these cases, quantitative methods may be helpful in providing confirmation on the significance of such abnormalities, and increase specificity.

The main limitation of the present study resides in the absence of an autopsy confirmation of the biomarker-based diagnoses, which would have first allowed to overcome possible shortcomings of having used different types of in vivo biomarkers as gold standard, even if amy-PET and CSF are generally considered interchangeable [2, 3]. Moreover, autopsy-based diagnosis would have allowed to detect possible cases of mixed pathology, whose presence in our sample may have resulted in an underestimation of the accuracy of FDG-PET in detecting brain amyloidosis. In fact, like in previous studies [21, 22, 35], we did not consider hypometabolism in AD-typical regions as an index of brain amyloidosis when there was more prominent hypometabolism elsewhere in the brain (as may be seen in the presence of co-pathology). We believe, though, that this risk was limited: none of our 12 false-negative cases showed significant PCTP hypometabolism in addition to hypometabolism outside of PCTP, reducing the probability that they were cases of mixed pathology. A second limitation was the lack of information about apolipoprotein E4 allele status, which has been shown to be associated with an AD-like metabolic pattern unrelated to amyloid deposition [53, 54] and might, therefore, account for some of our false-positive cases. Third, we are aware that several automated methods for the assessment of FDG-PET scans are available, and that our conclusions on INLAB and Cortex ID Suite are not generalizable to all semi-quantitative techniques. Fourth, our findings were obtained in a heterogeneous population of MCI/dementia referrals to a tertiary, university-based memory clinic, and might not be readily generalizable to other clinical settings. Finally, the procedure followed in the study for VIUI does not completely overlap with the real clinical routine, whereby raters are not blinded to clinical information. This deviation from clinical practice, however, has probably led to an underestimation, and not a misleading overestimation, of performance of qualitative rating, since access to patients’ clinical records would have probably increased accuracy.

Our study overall supports the usefulness of PCTP hypometabolism in identifying biomarker-confirmed amyloidopathy at the single-patient level, but, as already emerged from previous reports [18–21, 23, 24], is sensitive more than it is specific. Importantly, this evidence was achieved in a particularly challenging scenario, since our cohort included patients in various disease stages and with classical as well as atypical variants of AD. Qualitative rating of FDG scans showed particularly high sensitivity, but lower specificity than visual rating combined with semi-quantitative data. Asymmetric, left temporo-parietal hypometabolism associated with language deficits, in particular, tended to be mistaken for a marker of amyloid pathophysiology. As recommended by Nuclear Medicine and Neurology experts [25], resorting to automated methods in such cases, or in cases with ambiguous patterns, would increase the rate of clear-cut classifications and yield the best sensitivity–specificity trade off.

## Supplementary Information

Below is the link to the electronic supplementary material.

Supplementary file1 (DOCX 15 KB)
