# Supplementary material for: Validity of cingulate–precuneus–temporo-parietal hypometabolism for single-subject diagnosis of biomarker-proven atypical variants of Alzheimer’s Disease
Source: J Neurol. 2022 Mar 26;269(8):4440–51. doi: 10.1007/s00415-022-11086-y (PMC9293827; doi:10.1007/s00415-022-11086-y)
Supplement: Supplementary file 1 — Supplementary file1 (DOCX 15 KB) [file 415_2022_11086_MOESM1_ESM.docx]

**Supplementary table. Characteristics of cases unclassifiable (UN) by visual interpretation of uptake images and/or INLAB procedure. Rating by Cortex ID Suite was conclusive for all patients.**

|  | **Results of FDG-PET rating** | | | **Amyloid** | **Age** | **Syndrome** | **Months onset-** | **MMSE** | **Biomarker** | **Tau/Aβ** |
| --- | --- | --- | --- | --- | --- | --- | --- | --- | --- | --- |
|  | **VIUI** | **INLAB** | **Cortex ID** | **status** |  |  | **FDG PET** |  |  | **ratio** |
| **UN1** | UN | UN | True positive | A+ | 81 | AMN | 36 | 25 | PET | - |
| **UN2** | UN | UN | True positive | A+ | 72 | bFTD | 36 | 29 | CSF | 1.61 |
| **UN3** | UN | UN | True negative | A- | 70 | bFTD^a^ | 18 | 24 | CSF | 0.31 |
| **UN4** | UN | UN | False positive | A- | 71 | PNFA | 30 | 14 | CSF | 0.98 |
| **UN5** | UN | False negative | False negative | A+ | 79 | CBS^a^ | 24 | 16 | CSF | 1.32 |
| **UN6** | UN | False negative | True positive | A+ | 81 | bFTD | 24 | 28 | PET | - |
| **UN7** | UN | True negative | True negative | A- | 70 | bFTD^a^ | 12 | 25 | CSF | 0.21 |
| **UN8** | UN | True negative | True negative | A- | 70 | AMN | 36 | 21 | PET | - |
| **UN9** | UN | True negative | True negative | A- | 77 | AMN | 36 | 25 | CSF | 0.41 |
| **UN10** | UN | True negative | True negative | A- | 70 | DLB | 18 | 21 | CSF | 0.46 |
| **UN11** | UN | True negative | True negative | A- | 56 | SD | 12 | 27 | PET | - |
| **UN12** | UN | True negative | True negative | A- | 61 | PSP^a^ | 24 | 24 | CSF | 0.35 |
| **UN13** | UN | True negative | False positive | A- | 74 | PSP | 36 | 17 | PET | - |
| **UN14** | True positive | UN | False negative | A+ | 66 | AMN^a^ | 12 | 24 | PET | - |

^a^ Moderate impairment of speech

LEGEND: MMSE= MiniMental State Examination, AMN= Amnestic, bFTD= behavioural Frontotemporal Dementia, CSF= Cerebrospinal fluid, PNFA= Progressive Non-Fluent Aphasia, CBS= Corticobasal Syndrome, DLB= Dementia with Lewy Bodies, PSP= Progressive Supranuclear Palsy, SD= Semantic Dementia, VIUI= Visual Interpretation of Uptake Images
